# Triptolide mitigates radiation-induced pneumonitis via inhibition of alveolar macrophages and related inflammatory molecules

**DOI:** 10.18632/oncotarget.16456

**Published:** 2017-03-22

**Authors:** Chun Chen, Shanmin Yang, Mei Zhang, Zhenhuan Zhang, Steven B. Zhang, Bing Wu, Jinsheng Hong, Weijian Zhang, Jianhua Lin, Paul Okunieff, Lurong Zhang

**Affiliations:** ^1^ Department of Pharmacology, School of Pharmacy, Fujian Medical University, Fuzhou 350122, China; ^2^ Department of Radiation Oncology, University of Florida, Gainesville 32610, Florida, USA; ^3^ Fujian Platform for Medical Research at First Affiliated Hospital, Fujian Key Lab of Individualized Active Immunotherapy and Key Laboratory of Radiation Biology of Fujian Province Universities, Fuzhou 350005, China

**Keywords:** Triptolide, radiotherapy, pneumonitis, alveolar macrophage, inflammative molecules

## Abstract

Ionizing radiation-induced pulmonary injury is a major limitation of radiotherapy for thoracic tumors. We have demonstrated that triptolide (TPL) could alleviate IR-induced pneumonia and pulmonary fibrosis. In this study, we explored the underlying mechanism by which TPL mitigates the effects of radiotoxicity. The results showed that:

(1) Alveolar macrophages (AMs) were the primary inflammatory cells infiltrating irradiated lung tissues and were maintained at a high level for at least 17 days, which TPL could reduce by inhibiting of the production of macrophage inflammatory protein-2 (MIP-2) and its receptor CXCR2.

(2) Stimulated by the co-cultured irradiated lung epithelium, AMs produced a panel of inflammative molecules (IMs), such as cytokines (TNF-α, IL-6, IL-1α, IL-1β) and chemokines (MIP-2, MCP-1, LIX). TPL-treated AMs could reduce the production of these IMs. Meanwhile, AMs isolated from irradiated lung tissue secreted significantly high levels of IMs, which could be dramatically reduced by TPL.

(3) TPL suppressed the phagocytosis of AMs as well as ROS production.

Our results indicate that TPL mitigates radiation-induced pulmonary inflammation through the inhibition of the infiltration, IM secretion, and phagocytosis of AMs.

## INTRODUCTION

Ionizing radiation (IR) induced pneumonitis is a common and serious complication of radiotherapy for thoracic cancers, including lung cancer, esophageal cancer, breast cancer, and mediastinal tumors, and presents a major limiting factor for radiotherapy [1, 2]. Unfortunately, there is no FDA-approved drug that can prevent or mitigate radiation-induced pulmonary toxicity; however, our research has shown that triptolide (TPL), a small molecule purified from the herb *Tripterygium wilfordii Hook F* (also known as Thunder God Vine), has the potential to eventually fill this gap in the armamentarium and counteract radiation-induced pulmonary toxicity through its anti-inflammatory, immunosuppressive, and antitumor effects [3–7]. Our previous work showed that TPL alleviates radiation-induced pulmonary injury, including pneumonitis and fibrosis, in a mouse model [8, 9]. In the present study, we explored the underlying mechanism by which TPL mitigates the effects of radiotoxicity.

Radiation-induced pneumonitis features increased apoptosis of resident cells, recruitment of inflammatory cells, increased reactive oxygen species (ROS), and high levels of cytokines, chemokines, and growth factors mediating the interactions between multiple cell types [10–12]. Alveolar macrophages (AMs), which are innate immune cells in lung tissue, play a major role in inflammation and wound healing [13, 14]. Therefore, we examined the effects of TPL on pulmonary radiotoxicity by examining AM activities, such as migration, interactions with the lung epithelium, the production of IMs, phagocytosis, and ROS production.

## RESULTS

### AMs were the primary inflammatory cells in radiation-induced pneumonitis

**Figure 1:**
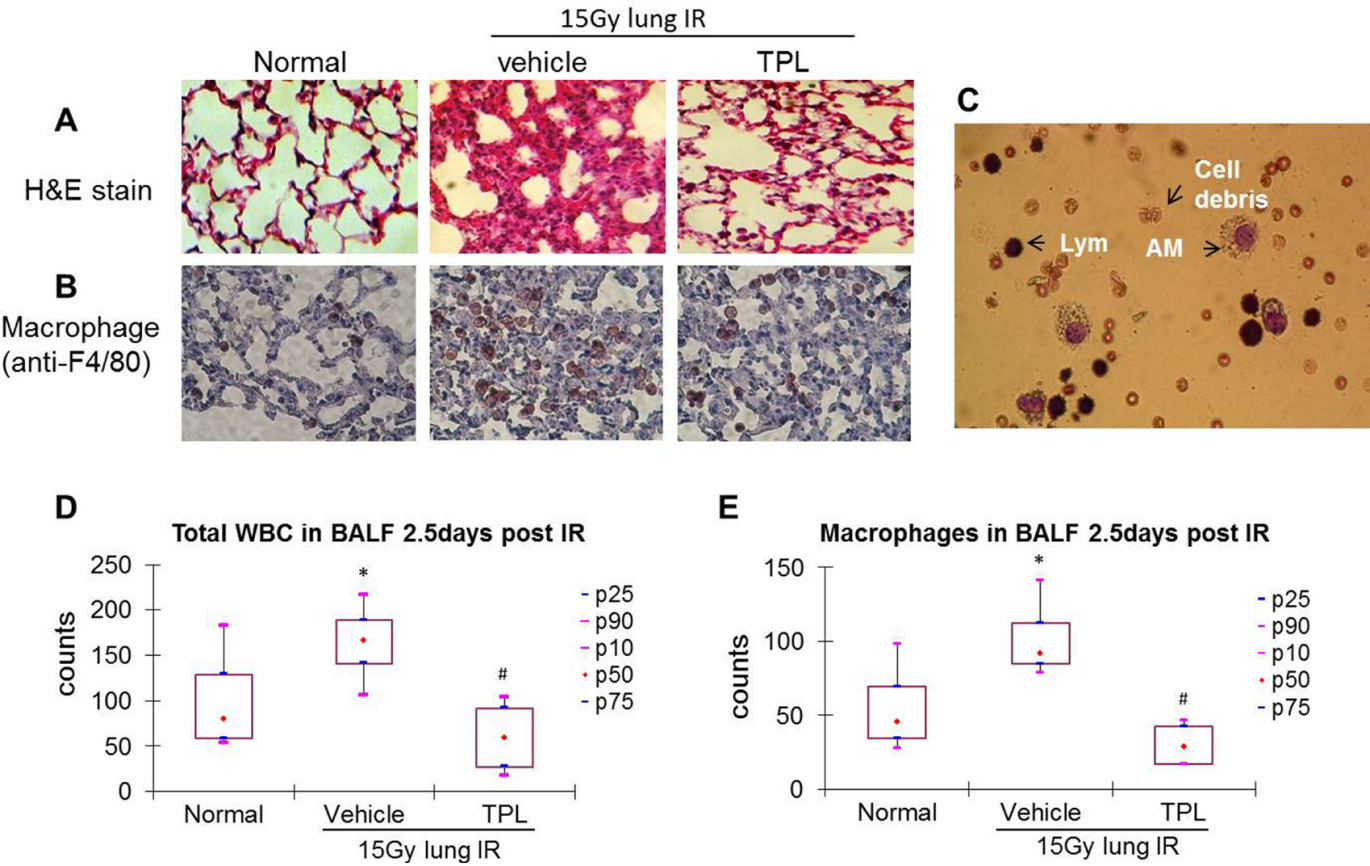
TPL reduced AMs in lung tissue and BALF at 2.5 days after thoracic irradiation Paraffin-embedded sections were prepared from lung tissues collected 2.5 days post thoracic irradiation. (**A**) H&E stain (×100). (**B**) Immunohistochemistry using anti-F4/80 primary antibody and colored by AEC kit (×400). (**C**) Differential cell count in BALF was carried out on Wright-Giemsa stained cytospin smears. AMs: alveolar macrophages; Lym: alveolar lymphocytes. Cells in BALF were counted under a microscope (**D** and **E**). *P < 0.05 vs. Normal control group; ^#^*P* < 0.05 vs. IR Vehicle control group. TPL: TPL 0.25 mg/kg IV.

**Figure 2:**
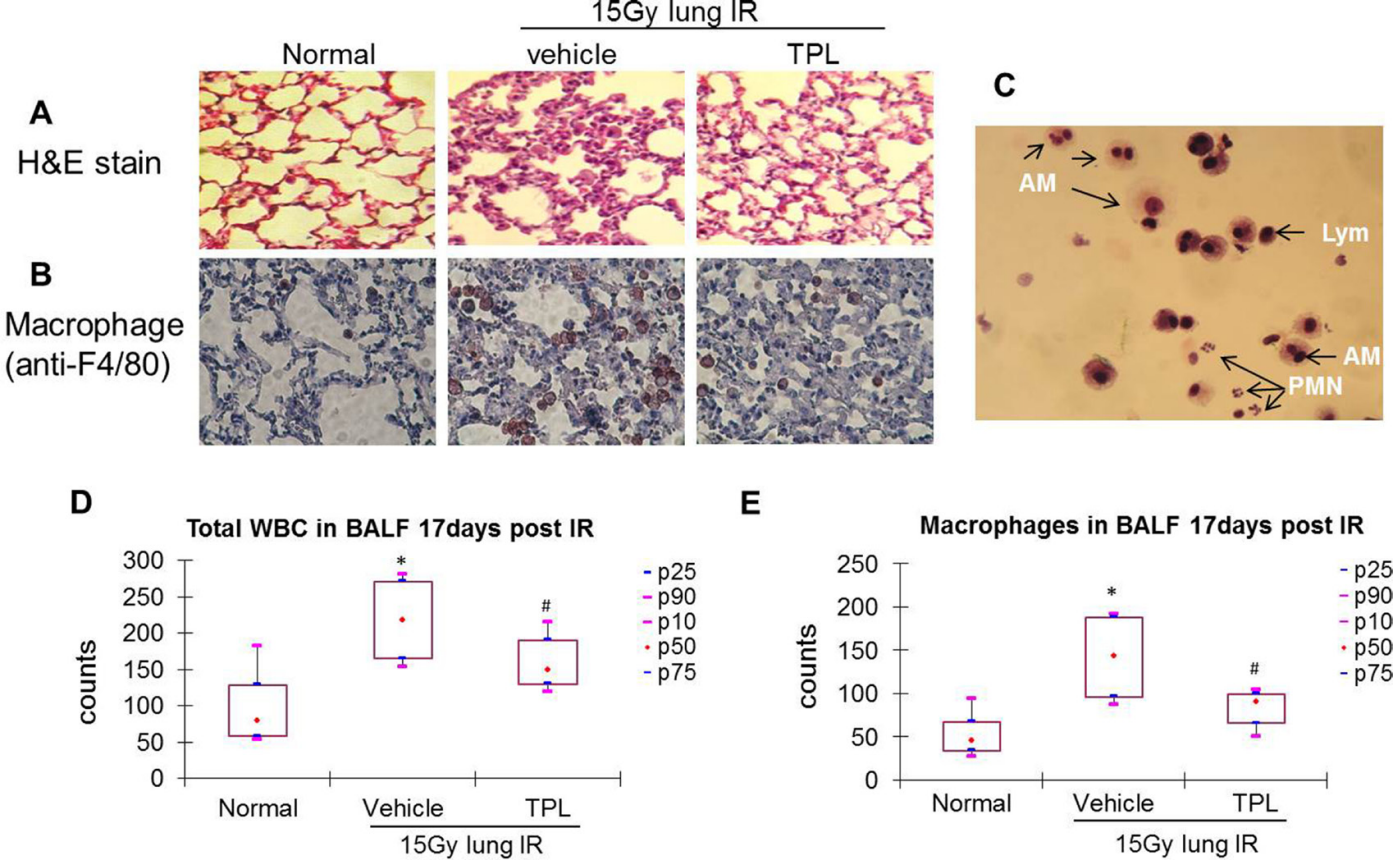
TPL reduced AMs in lung tissue and BALF at 17 days after thoracic irradiation Paraffin-embedded sections were prepared from lung tissues collected 17 days post thoracic irradiation. (**A**) H&E stain (×100). (**B**) Immunohistochemistry using anti-F4/80 primary antibody and colored by AEC kit (×400). (**C**) Differential cell count in BALF was carried out on Wright-Giemsa stained cytospin smears. AMs: alveolar macrophages; Lym: alveolar lymphocytes; PMNs: alveolar neutrophils. Cells in BALF were counted under a microscope (**D** and **E**). *P < 0.05 vs. Normal control group; ^#^*P* < 0.05 vs. IR Vehicle control group. TPL: TPL 0.25 mg/kg IV.

Acute phase (2.5 days post-IR) and sub-acute phase (17 days post-IR) radiation-induced pneumonitis samples were collected and studied. In the acute phase, the infiltration of inflammatory cells and exudates in irradiated mice led to septal thickening, indicating pneumonitis (HE staining, Figure 1A). Macrophages increased among the infiltrating cells, as evidenced by anti-F4/80-positive immunohistochemical (IHC) staining (Figure 1B). A similar pattern was seen in sample sets from mice in the sub-acute phase (Figure 2A and 2B).

In the acute phase, infiltrated cells were dominated by lymphocytes and AMs, with few neutrophils or dead epithelial cells (Figure 1C), in bronchoalveolar lavage fluid (BALF) samples. Total leukocyte and AM levels in BALF were elevated by ∼2-fold in irradiated mice as compared to normal control mice (Figure 1D and 1E). This pattern was altered in the sub-acute phase in which the neutrophils, AMs, and lymphocytes were dominant in BALF (Figure 2C), yet the total leukocyte and AM levels were also elevated by ∼2-fold in irradiated mice as compared to controls (Figure 2D and 2E). These results demonstrate that AMs are the primary inflammatory cells present in radiation-induced pneumonitis and that they continue elevating throughout the acute and sub-acute phases.

### Anti-inflammatory effect of TPL was accompanied by suppression of infiltrating AMs

As a potent anti-inflammatory agent, TPL (at a low dose of 0.25 mg/kg) suppressed radiation-induced pneumonitis (Figure 1A and 2A) with a significant reduction of AMs in irradiated lung tissue (Figure 1B and 2B). This was also reflected in total leukocyte (Figure 1D and 2D) and AM (Figure 1E and 2E) levels in the BALF of irradiated mice compared to irradiated vehicle controls. These results suggest that TPL mitigates radiation-induced pneumonitis by suppressing infiltrated AMs.

### The suppression of AMs’ migration by TPL was related to MIP-2 and its receptor CXCR2

**Figure 3:**
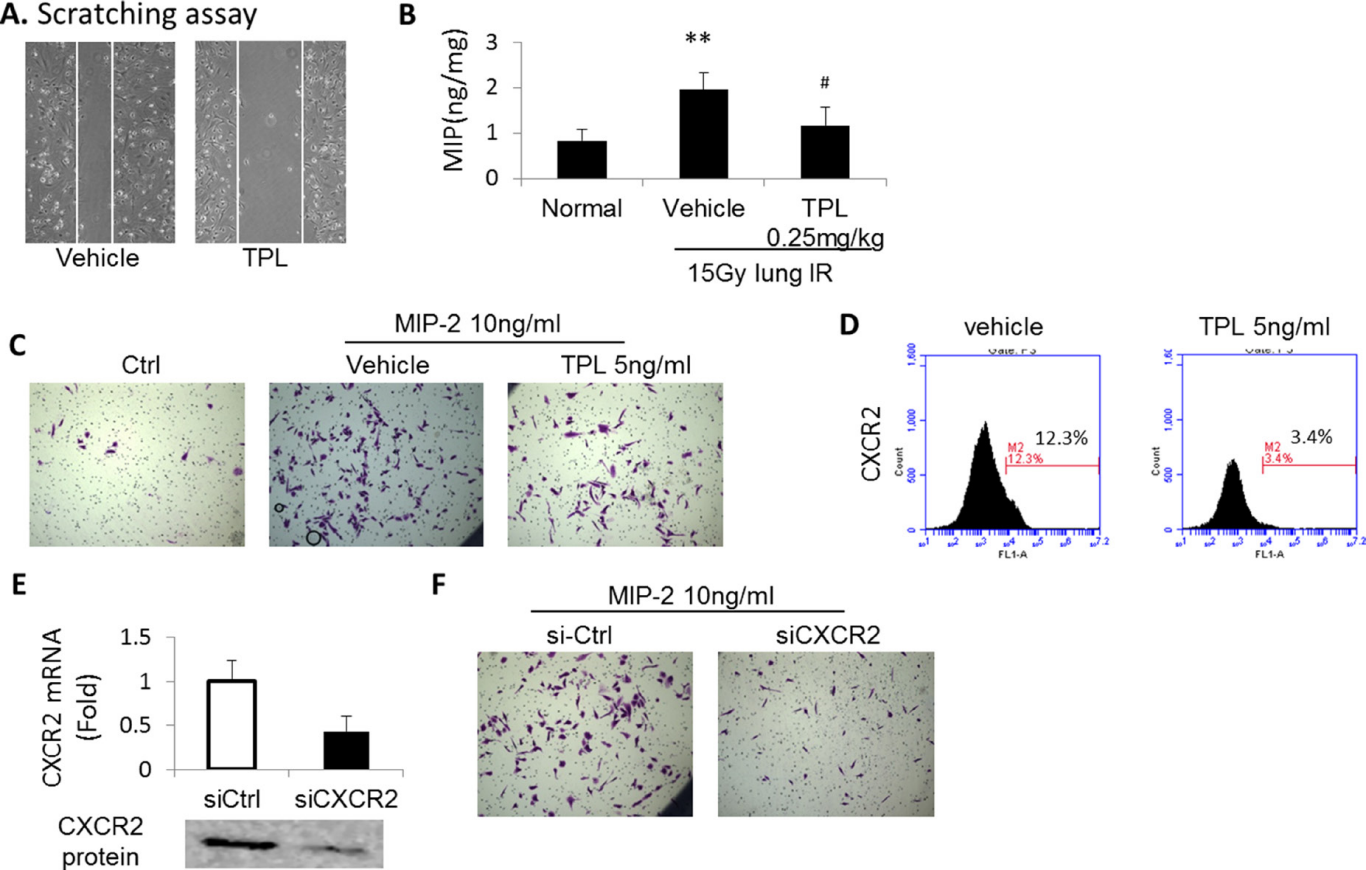
TPL downregulated chemokines of AMs and inhibited the migration of AMs (**A**) Scratch-wound assay was performed on MH-S cells treated with vehicle or TPL. (**B**) The level of MIP-2 in lung tissue at 2.5 days after thoracic irradiation. **P < 0.01 vs. Normal control group; ^#^*P* < 0.05 vs. IR Vehicle control group. (**C**) AM migration assayed by transmembrane assay. The TPL group was pretreated with TPL, and then induced by MIP-2. (**D**) The expression of CXCR2 on primary AMs at 2.5 days after thoracic irradiation and /or TPL treatment. (**E**) CXCR2 siRNA effectively knocked down the mRNA level and protein expression of CXCR2 in MH-S cells. (**F**) The migration capacity of MH-S-CXCR2-siRNA cells assayed by transmembrane assay.

We performed the scratch-wound assay to determine if TPL affected macrophages in the blood migrating into irradiated lung. This assay detects “sheet migration,” which occurs in diverse processes, such as cancer metastasis, tissue injury, and macrophage invasion [15–17]. Our results showed that TPL suppressed MH-S cells migration (Figure 3A).

Our previous study showed that macrophage inflammatory protein-2 (MIP-2), which plays an important role in inflammatory cell migration [18], was the dominant chemokine in irradiated lung tissue [9]. As such, we examined the effect of TPL on MIP-2 production. The lysate obtained from irradiated lung tissues was measured with a MIP-2 ELISA kit. As Figure 3B shows, TPL reduced MIP-2 production.

To determine if TPL also suppressed MIP-2-induced migration of macrophages, we performed a transwell migration assay. When recombinant MIP-2 (10 ng/ml) was added to the bottom of the transwells, MH-S macrophage migration surged to a high level that was suppressed by TPL at 5 ng/ml (Figure 3C), indicating that TPL reduces the responsiveness of MH-S macrophages to MIP-2.

We also suspected that this reduction might be related to TPL's effect on the MIP-2 receptor CXCR2. Thus, MH-S cells were treated with TPL (5 ng/ml) for 2 days, and the level of cell-surface CXCR2 was examined with FCM. Figure 3D shows that the expression of CXCR2 was reduced from 12.3% to 3.4% following TPL treatment. When CXCR2 was knocked down with siRNA (Figure 3E), the migration of MH-S cells was back to the same pattern as TPL treatment (Figure 3F vs. Figure 3C), suggesting that TPL reduced responsiveness of MH-S macrophage to MIP-2 via suppression of CXCR2 expression, and thereby, decreasing the infiltrated AMs in the irradiated lung.

### TPL downregulated inflammatory molecules in radiation-induced pneumonitis through the inhibition of macrophages

**Figure 4:**
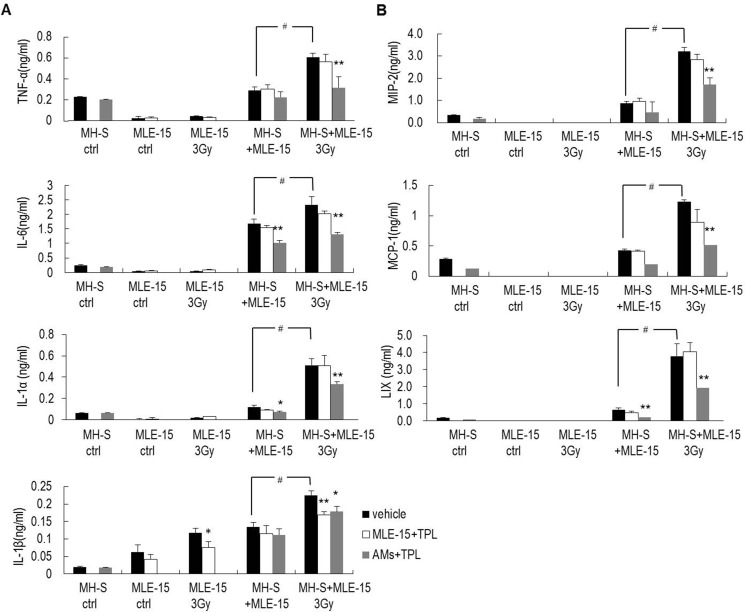
TPL downregulated cytokines and chemokines secreted by AMs under the insult of irradiated MLE-15 cells (**A**) Inflammatory factors, such as TNF-α, IL-6, IL-1α, and IL-1β and (**B**) chemokines, such as MIP-2, MCP-1, and LIX in co-culture medium were detected by ELISA. *P < 0.05, **P < 0.01 vs. vehicle group; ^#^*P* < 0.01. Note: MLE-15+TPL: MLE-15 cells pretreated with 5 ng/ml of TPL; AMs+TPL: MH-S cells pretreated with 5 ng/ml of TPL.

Radiation triggers activation of the IM network (including cytokines and chemokines) via the interaction between the lung epithelium and macrophages [19]. To explore the effect of TPL on this interaction, the production of a panel of IMs after co-culture of MH-S macrophages and irradiated MLE-15 lung epithelium were measured with ELISA kits. When MH-S macrophages and MLE-15 lung epithelium were cultured separately, each produced very low levels of IMs, including TNF-α, IL-6, IL-1α, MIP-2, MIP-1α, LIX, even following irradiation (with the notable of exception of IL-1β, which increased following 3-Gy irradiation). However, when these cells were co-cultured, IMs significantly increased, especially when the nonirradiated macrophages were able to sense the stress signal released from the irradiated MLE-15 and the cells worked together to produce all seven IMs (TNF-α, IL-6, IL-1α, MIP-2, MIP-1α, LIX, and IL-1β) in a significantly increased manner. This surge was reduced by pre-treatment of MH-S macrophages with TPL, but not by pre-treatment of MLE-15 cells with TPL (Figure 4), suggesting that macrophages play a more important role than epithelial cells in the production of the IMs that affect the pathophysiological process of radiation-induced pulmonary toxicity.

**Figure 5:**
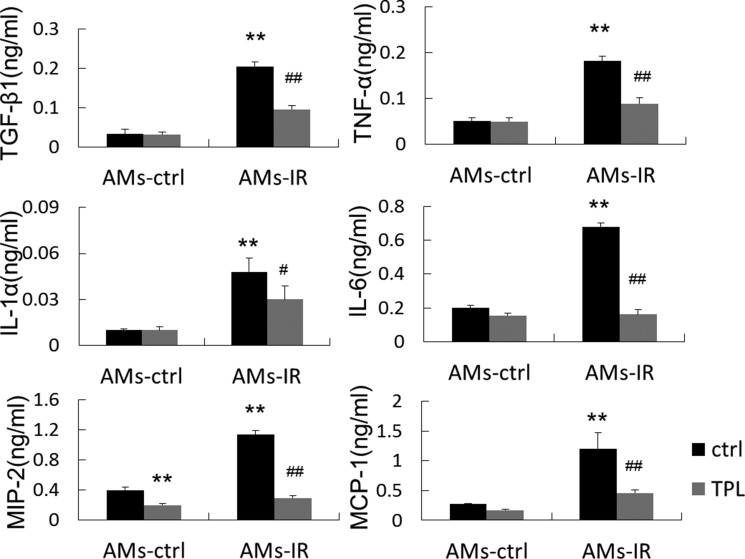
TPL reduced cytokine production in AMs from lung tissue at 2.5 days after thoracic irradiation Isolated AMs were treated with or without TPL (5 ng/ml) for 24 h, and the supernatants were collected and assayed by ELISA (*n* = 5, in duplicate). **P < 0.01 vs. ctrl of AMs-ctrl. ^#^*P* < 0.05, ^##^*P* < 0.01 vs. ctrl of AMs-IR.

To further confirm that the alteration of IMs in the MH-S macrophage cell line was true in freshly isolated primary AMs, we obtained AMs from normal control mice (AMs-Ctrl) and thoracic irradiated mice (AMs-IR) treated with or without TPL. The production of a panel of key proinflammatory/profibrotic cytokines, including IL-1α, IL-6, TNF-α, MIP-2, MCP-1, and TGF-β1, occurred in AMs-Ctrl regardless of TPL treatment. IMs surged several times in AMs-IR, but this was suppressed by TPL (Figure 5), suggesting that the study result obtained from the MH-S macrophage cell line reflected the behavior of primary AMs from irradiated mice.

### TPL inhibited the phagocytosis of AMs and reduced the production of ROS

**Figure 6:**
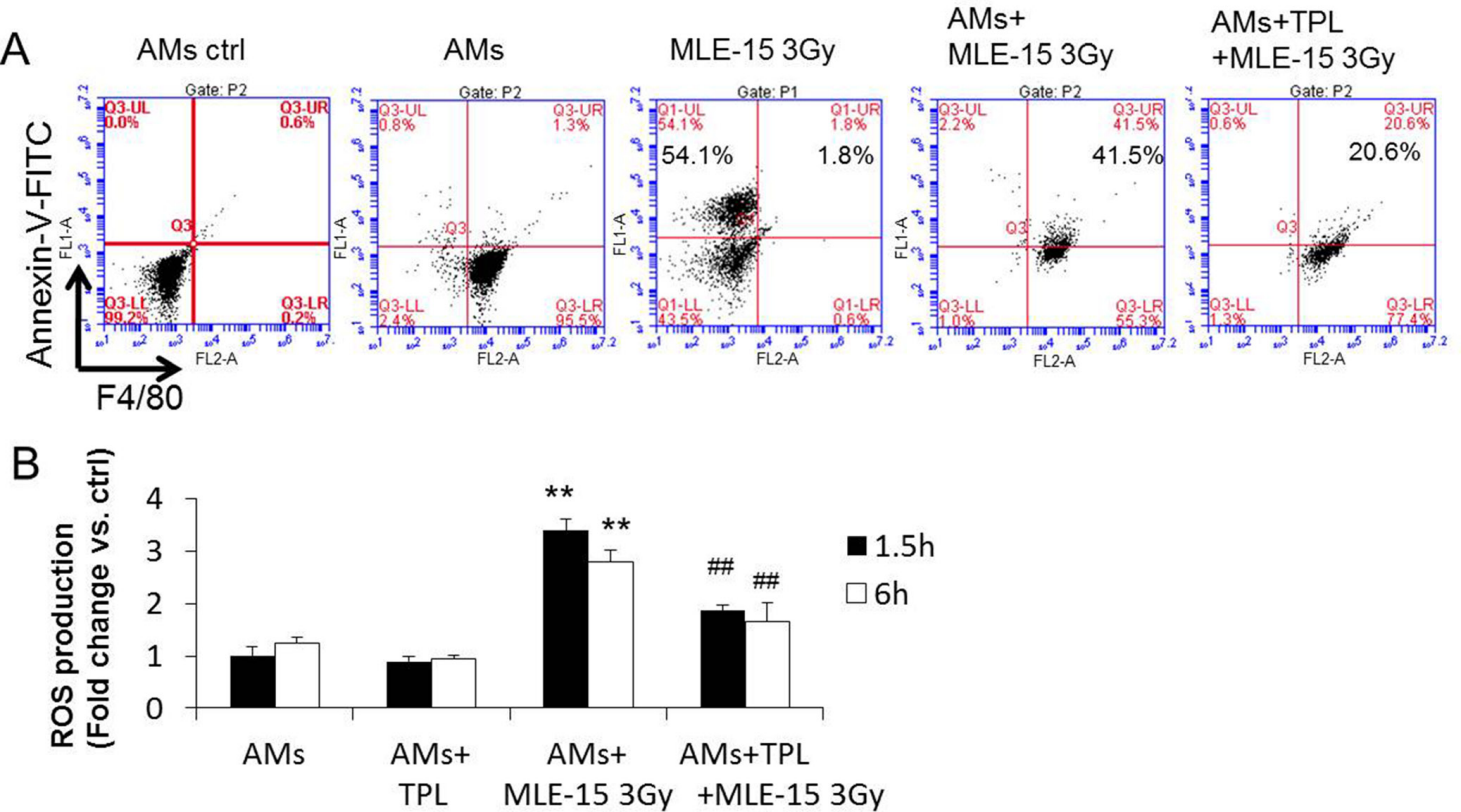
TPL inhibited phagocytosis of AMs and reduced the production of ROS by AMs (**A**) Primary AMs phagocytizing apoptotic MLE-15 cells were quantitatively analyzed by flow cytometry (see detail in methods), phagocytic AMs were double-positive cells for PE (labeled F4/80) and FITC (labeled apoptotic cell), AMs ctrl stained with isotype antibody. (**B**) Intracellular ROS levels were measured using DCFH-DA. **P < 0.01 vs AMs group; ^##^*P* < 0.01 vs. AMs+MLE-15 3 Gy group.

Radiation causes cell death and apoptosis. The removal of the dead/apoptotic cells by phagocytosis of AMs has two effects: (1) to clean up the alveoli; and (2) to trigger the subsequent inflammatory response, such as the production of IMs and ROS. To determine if TPL could suppress the phagocytosis of AMs, MLE-15 epithelial cells were irradiated (3 Gy) to induce apoptosis and then 3 h later stained with Annexin V. AMs were labeled with PE-anti-F4/80. Two types of labeled cells were co-cultured for 1.5 h to allow AMs to phagocytose and clean up the apoptotic MLE-15 cells. Indeed, 54% of irradiated MLE-15 cells were apoptotic, and most of the apoptotic MLE-15 cells (41.5%) were phagocytosed by AMs. When AMs were pre-treated with TPL, their phagocytosis was reduced to 20% (Figure 6A), suggesting that TPL reduced radiation-induced pulmonary injury by suppressing the phagocytosis of AMs to avoid the subsequent inflammatory cascade [20, 21].

During phagocytosis, AMs could generate and release ROS, triggering further pulmonary injury [22]. The reduction of ROS in AMs is likely to reduce radiation-induced pneumonitis [23]. Therefore, we examined the effect of TPL on the production of ROS when irradiated MLE-15 cells were co-cultured with AMs for 1.5 h. Figure 6B shows that when AMs were cultured alone with or without TPL, they produced limited ROS. However, when AMs were co-cultured with 3-Gy irradiated MLE-15 cells, the ROS surged ∼3 times higher; this surge was reduced in AMs pre-treated with TPL, indicating that TPL suppressed the ROS during phagocytosis.

## DISCUSSION

Triptolide is a potent anti-inflammatory agent [3, 24] that can alleviate radiation-induced inflammation. However, its mechanisms of action are unclear. Since macrophages play a major role in radiation-induced pulmonary inflammation and fibrosis, our study focused on the interactions of TPL with AMs, lung epithelial cells, and their related molecules following irradiation. This work produced the following results.

(1) During the acute phase (2.5 days post-IR), macrophages were the primary infiltrating cells. In BALF, AMs, lymphocytes, and dead cell debris dominated. In the transition into the sub-acute phase (17 days post-IR), AMs remained as increased infiltrated cells even the cellular debris was removed.

(2) The suppression of macrophage migration by TPL was partially related to the reduction of CXCR2 expression and, thus, inhibition of MIP-2-mediated migration of macrophages.

(3) The interaction of irradiated lung epithelium with macrophages led to the induction of a panel of IMs (TNF-α, IL-6, IL-1α, MIP-2, MCP-1 and LIX), which was suppressed by TPL.

(4) IL-1α, IL-6, TNF-α, MIP-2, MCP-1, and TGF-β1 production surged in freshly harvested AMs from irradiated lung tissue, which could be reduced with TPL.

(5) TPL inhibited the phagocytosis of AMs and the corresponding surge of ROS.

Taken together, TPL reduces pulmonary inflammation by suppressing the migration and phagocytosis of lung macrophages as well as the production of IMs and ROS.

Anti-inflammatory therapies primarily work by inhibiting macrophage infiltration and pro-inflammatory cytokines. Our study showed that the key targeting molecules of macrophage infiltration might be MIP-2 and its receptor CXCR2. TPL could reduce their expression and suppress the responsiveness of the macrophage to the chemokine. This dual suppression of chemoattractive effects and production of a panel of IMs (cytokines and chemokines) may account for TPL's potent suppression of radiation-induced inflammation. We found that the interaction between macrophages and radiation-damaged epithelial cells was the key IM surges (i.e., when infiltrated macrophages encounter the radiation-damaged epithelial cells, the IMs and ROS surge, thereby indicating that the macrophages, and not radiation-damaged epithelial cells, play a major role in the cascade of radiation-induced inflammation, which TPL suppresses).

We [25] and others [26, 27] have found that an acute radiation-induced reaction (e.g., exudate, infiltrated WBC, cytokine storm) occurs in 1-3 days. Therefore, day 2.5 after irradiation was selected as the first time point (i.e., the acute phase). A second surge that occurred 2-3 weeks after irradiation might be attributable to a subsequent immune response; thus, we utilized samples from day 17 to determine alterations in the sub-acute phase. In *in vitro* studies, primary AMs were isolated from lung tissue at 2.5 days after thoracic irradiation; macrophages (MH-S cell line) were co-cultured with irradiated alveolar epithelial cells and incubated for 24 hours. Thus, the detected cytokine changes mainly reflected pathophysiological changes in the acute phase of radiation-induced pneumonitis.

Based on their location in the lung, macrophages can be divided into the alveolar and interstitial subtypes. Despite some phenotypic differences [28, 29], these subpopulations function similarly during acute phase and also share similar trends in surface marker and gene expression [29].

Based on phenotype and function, macrophages can also be classified into classically activated macrophages and alternatively activated macrophages [30]. The classically activated macrophages express inducible nitric oxide synthase (iNOS) and produce TNF-α, IL-1α, and IL-6 to stimulate inflammatory responses, whereas alternatively activated macrophages express arginase type 1 (Arg-1) and mannose receptor, produce TGF-β1 and PDGF, and are involved in tissue repair [31]. Much of this classification is based on *in vitro* studies, *in vivo* studies have shown that macrophages share some overlapping features [29]. We found that irradiated alveolar macrophages had some functions of both subtype macrophages, which not only express TNF-α, IL-1α, IL-6, and TGF-β1 but also produce ROS that might damage lung tissues.

A study of Sprague-Dawley rats showed that 40 min after TPL administration (0.6 mg/kg, IV), the plasma level of TPL was still higher than 10 ng/ml [32]. Although a report of pharmacokinetics of TPL in C57BL/6 mice is lack, logically the administration of TPL (0.25 mg/kg, IV) would reach a plasma concentration higher than 2 ng/ml. Furthermore, our previous *in vitro* dose-dependent study with 2, 5 and 10 ng/ml TPL indicated that all three doses inhibited cytokine production, with the most striking effect at 10 ng/ml [9]. To ensure an obvious effect while avoiding toxicity as well as to utilize concentrations close to those needed *in vivo*, we ultimately selected 5 ng/ml.

Technically, this study set up a unique co-culture system to mimic the cell-cell interaction of type II pneumocytes with macrophage *in vivo*, which could be utilized for study the effects of anti-lung inflammation agents.

A large body of data, including ours, confirms that TPL is a potent anticancer and anti-inflammatory agent [33–35]. Although the high toxicity of TPL, including liver and kidney toxicity and myelosuppression [36], has limited its clinical use thus far, several promising solutions have been developed to increase its therapeutic potential. Zhang L et al synthesized and *in vitro* and *in vivo* tested a nanodrug carrier system (γ-PGA-l-PAE-TPL) that delivers a less toxic form of TPL [37]. Fidler et al developed MRx102, a TPL lipophilic derivative, that is 20-fold to 60-fold safer than TPL in rat models yet maintains its anticancer activity at nanomolar concentrations [34, 38]. Once the toxicity of TPL is decreased, the use of TPL in anti-cancer and anti-inflammation will become much practicable in near future.

## MATERIALS AND METHODS

### Mouse model of thoracic irradiation

The whole chest irradiation of C57BL/6 mice (female, 8 weeks old, Shanghai Slac Laboratory Animal Co., Ltd. [SCXK (hu) 2012-0002], China) was performed as previously described [9]. The irradiated mice were randomly divided into groups (10 mice/group) and subjected to either (1) saline as a vehicle control or (2) TPL (0.25 mg/kg, IV, 0.2 ml/mouse, twice per week) 24 hours after thoracic irradiation. Non-irradiated mice were used as age controls. All animal studies and procedures were approved by Institutional Animal Welfare & Ethics Committee of Fujian Medical University (Fuzhou, China). The dose of TPL used in mice was determined by previous work [9].

### Sample collection and counting of AMs

At 2.5 days (1 treatment) and 17 days (5 treatments) post-irradiation, the mice samples of lung tissues and BALF were collected and prepared as described previously [8]. The BALF samples were centrifuged at 1500 rpm for 5 min, and the cell pellet were smeared in a single cell layer on microscope slides. A Wright-Giemsa stain was used to differentiate between blood cell types. The cells were counted continuously in the head, middle, and tail of each smear with 20 high-power fields.

### Culture of primary alveolar macrophages

Primary AMs were isolated from the BALF obtained from C57BL/6 mice. The AM culturing method was described previously [8].

### Co-culture of AMs with irradiated alveolar epithelial cells

Murine AM cell line MH-S and murine alveolar epithelial cell line MLE-15 were obtained from the Shanghai Cell Bank of the Chinese Academy of Sciences. Corning transwell plates (Sigma-Aldrich, St. Louis, MO, USA) with 0.4-μm pore polycarbonate membrane inserts were used for co-culture to allow the exchange of secreted biofactors between two types of cells while restricting direct cellular contact. MH-S cells were cultured in transwell inserts of a 12-well culture plate, while MLE-15 cells were cultured in another 12-well plate. Immediately before MLE-15 cells were irradiated, both media were changed to UltraDOMA-PF (Lonza Cat No.15-727D, Walkersville, MD, USA). After MLE-15 irradiation, the inserts containing AMs were immediately transferred to MLE-15 plates, and co-cultured for 24 hours. For TPL treatment, AMs and MLE-15 cells were pre-treated with TPL (5 ng/ml) for 6 hours before the co-culture. The cultured media were collected and used for cytokine/chemokine assay, as described below.

### ELISA for the different cytokines and chemokines

An enzyme-linked immunosorbent assay (ELISA) was performed to quantify cytokines and chemokines. The lung homogenates and the culture supernatants were collected, and the assays were performed with DuoSet kits (R&D Systems, Minneapolis, MN, USA) according to the manufacturer's instructions.

### Transwell migration assay

MH-S cells (2 × 10^4^/well) treated with or without TPL (5 ng/ml) for 6 h were seeded on the 8.0-μm pore membrane of the transwell, and no-serum RPMI 1640 with or without MIP-2 (10 ng/ml) was placed in the lower chamber. After incubating for 12 h at 37°C with 5% CO_2_, macrophages that had migrated through the membrane were stained with crystal violet and quantified using light microscopy.

### siRNA inhibition of CXCR2

The procedure of siRNA inhibition in MH-S cells was described previously [8]. si-m-CXCR2 (RiboBio Co., Ltd., Guangzhou, China) was used to knock down CXCR2 in MH-S cells, with noncoding siRNA as control. One of 3 siCXCR2 duplexes efficiently blocked CXCR2 expression.

### Immunohistochemical staining for AMs

Lung tissue slides were routinely rehydrated. Hematoxylin and eosin stains were conducted. Immunohistochemical staining for AMs was carried out as previously described [8].

### Macrophage phagocytosis assay

Phagocytosis of apoptotic cells by AMs was quantified using a flow cytometric assay [39]. MLE-15 cells were irradiated at 3 Gy, and 3 hours later the cells were collected and incubated with Annexin-V-FITC (Promega, USA) for 20 min in the dark. The stained MLE-15 cells were then added to the culture wells of primary AMs. After 30 min of incubation at 37°C/5% CO_2_, the medium was removed by gentle pipetting to minimize agitation of the sediment cells. The cells were collected and stained with PE-F4/80 antibodies (BioLegend, San Diego, CA, USA). Samples were assayed immediately by Accuri C6 Flow Cytometer^®^ and CFlow^®^ software (BD Biosciences, USA). All steps after Annexin-V-FITC staining were performed in dark conditions.

### Determination of ROS production

Primary AMs were cultured alone or with 3 Gyirradiated MLE-15 for direct cell-cell contact in UltraCULTURE^TM^ medium for different times (1.5 hr and 6 hr). The intracellular ROS levels were measured using 2, 7-dichlorodihydrofluorescein diacetate (DCFH-DA, Sigma-Aldrich) and examined with a fluorescence microplate reader as previously described [8]. The ROS values were expressed as fold changes compared to the controls.

### Statistical analysis

All statistical analyses were conducted with SPSS 19.0 software. Data was analyzed with one-way ANOVA and post-hoc multiple comparisons with Tamhane's T2. An unpaired Student *t*-test was used for single experimental and control groups. Differences with *P* values < 0.05 were considered significant.
